# The Palliation of Unresectable Pancreatic Cancer: Evolution from Surgery to Minimally Invasive Modalities

**DOI:** 10.3390/jcm14144997

**Published:** 2025-07-15

**Authors:** Muaaz Masood, Shayan Irani, Mehran Fotoohi, Lauren Wancata, Rajesh Krishnamoorthi, Richard A. Kozarek

**Affiliations:** 1 Division of Gastroenterology and Hepatology, Center for Digestive Health, Virginia Mason Franciscan Health, Seattle, WA 98101, USA; muaaz.masood@commonspirit.org (M.M.); shayan.irani@commonspirit.org (S.I.); rajesh.krishnamoorthi@commonspirit.org (R.K.); 2 Department of Radiology, Virginia Mason Franciscan Health, Seattle, WA 98101, USA; mehran.fotoohi@commonspirit.org; 3 Department of Surgery, Virginia Mason Franciscan Health, Seattle, WA 98101, USA; lauren.wancata@commonspirit.org; 4 Center for Interventional Immunology, Benaroya Research Institute, Virginia Mason Franciscan Health, Seattle, WA 98101, USA

**Keywords:** palliation, pancreatic cancer, obstructive jaundice, gastric outlet obstruction, cholecystitis, refractory pain

## Abstract

Pancreatic cancer is an aggressive malignancy, with a current 5-year survival rate in the United States of approximately 13.3%. Although the current standard for resectable pancreatic cancer most commonly includes neoadjuvant chemotherapy prior to a curative resection, surgery, in the majority of patients, has historically been palliative. The latter interventions include open or laparoscopic bypass of the bile duct or stomach in cases of obstructive jaundice or gastric outlet obstruction, respectively. Non-surgical interventional therapies started with percutaneous transhepatic biliary drainage (PTBD), both as a palliative maneuver in unresectable patients with obstructive jaundice and to improve liver function in patients whose surgery was delayed. Likewise, interventional radiologic techniques included the placement of plastic and ultimately self-expandable metal stents (SEMSs) through PTBD tracts in patients with unresectable cancer as well as percutaneous cholecystostomy in patients who developed cholecystitis in the context of malignant obstructive jaundice. Endoscopic retrograde cholangiopancreatography (ERCP) and stent placement (plastic/SEMS) were subsequently used both preoperatively and palliatively, and this was followed by, or undertaken in conjunction with, endoscopic gastro-duodenal SEMS placement for gastric outlet obstruction. Although endoscopic ultrasound (EUS) was initially used to cytologically diagnose and stage pancreatic cancer, early palliation included celiac block or ablation for intractable pain. However, it took the development of lumen-apposing metal stents (LAMSs) to facilitate a myriad of palliative procedures: cholecystoduodenal, choledochoduodenal, gastrohepatic, and gastroenteric anastomoses for cholecystitis, obstructive jaundice, and gastric outlet obstruction, respectively. In this review, we outline these procedures, which have variably supplanted surgery for the palliation of pancreatic cancer in this rapidly evolving field.

## 1. Introduction

**Figure 1:**
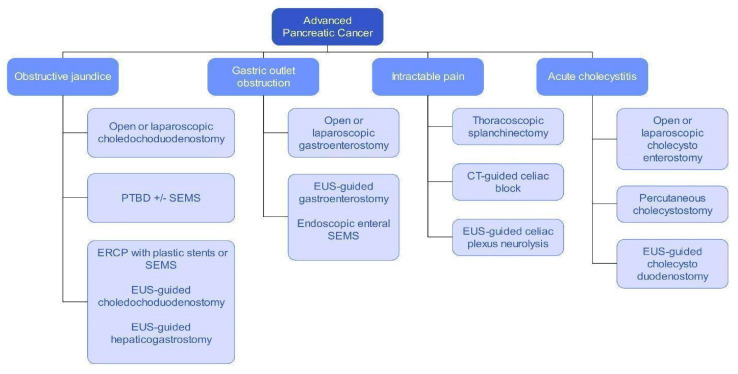
A schematic representation of the multimodal management of advanced pancreatic cancer sequelae. Abbreviations: CT = computed tomography, ERCP = endoscopic retrograde cholangiopancreatography, EUS = endoscopic ultrasound, SEMS = self-expandable metal stents, PTBD = percutaneous transhepatic biliary drainage.

Pancreatic cancer is the third leading cause of cancer-related death in the US after lung cancer and colon cancer. It is projected to become the second leading cause of cancer-related death by 2030. Pancreatic cancer is often diagnosed at an advanced stage when there is local invasion or distal metastases. The five-year survival rate for pancreatic cancer has increased from 7% to approximately 13.3% over the past 10 years according to Surveillance, Epidemiology, and End Results (SEER) program data (Source: American Cancer Society; Cancer Facts & Figures 2025; Atlanta: American Cancer Society; 2025). The majority of pancreatic cancers are located in the head of the pancreas and can result in biliary and gastric outlet obstruction. Over half of patients have metastases at the time of diagnosis. Liver metastases are the most common, but pancreatic cancer may also spread to local vasculature or metastasize to the lung, peritoneum, and bones, which precludes surgery. Approximately 15–20% of pancreatic tumors are resectable at diagnosis (Source: American Cancer Society; Cancer Facts & Figures 2024; Atlanta: American Cancer Society; 2024). The current standard for resectable pancreatic cancer most commonly includes neoadjuvant chemotherapy prior to a curative resection [1,2]. Advanced pancreatic cancer refers to pancreatic cancer that is unresectable or with metastases, which often results in complications (Figure 1). Advanced pancreatic cancer may lead to several sequelae including obstructive jaundice, gastric outlet obstruction (GOO), intractable pain, and acute cholecystitis, which can significantly affect the quality of life of patients. The timing of systemic therapy is based on a multidisciplinary discussion in a pancreatic tumor board. In this review, we describe the evolution of palliation in advanced pancreatic cancer from surgery to minimally invasive modalities involving interventional radiology and advanced endoscopy.

## 2. Methods

A narrative review of the literature regarding the palliation of unresectable pancreatic cancer was conducted. PubMed computerized search and Google Scholar were utilized to identify articles with the following title or keywords: “advanced pancreatic cancer”, “unresectable pancreatic cancer”, “obstructive jaundice”, “gastric outlet obstruction”, “intractable pain”, and “acute cholecystitis”. Articles were excluded if they were not available in English or as full texts. Duplicate articles or articles that were unrelated to the topic were also excluded. Systematic reviews, meta-analyses, and randomized controlled trials were assigned a high priority. This article does not contain novel studies with human participants or animals that were conducted by any of the authors. The authors contributed original radiographic and endoscopic images, which serve to highlight the points outlined in the manuscript. All authors reviewed articles within their discipline. The manuscript and images were independently reviewed and revised by all authors.

### 2.1. Surgical Palliation

Surgery in most patients with advanced pancreatic cancer has historically been palliative in nature. The latter interventions include open or laparoscopic bypass of the bile duct or stomach in cases of obstructive jaundice or GOO, respectively. A study of 42 patients, who underwent either palliative gastrojejunostomy or hepaticojejunostomy, reported severe postoperative morbidity and mortality rates and emphasized patient selection for acceptable postsurgical outcomes [3]. Espat et al. noted that the practice of routine prophylactic bypass procedures was not supported and should only be performed in patients with obstructive jaundice who do not undergo endoscopic stent placement or in patients with GJ and confirmed GOO [4].

Lyons et al. demonstrated that prophylactic duodenal, biliary, and dual bypasses in patients with unresectable pancreatic cancer during index laparotomy did not result in fewer invasive procedures or reduce the number of hospital days [5]. The patients who underwent biliary bypass seldom required treatment for biliary obstruction. Additionally, patients who had duodenal bypass seldom required interventions for GOO. The double bypass group was associated with just as many postoperative interventions and accrued hospital days as the duodenal bypass and biliary bypass groups [5]. In contrast, a prospective randomized controlled trial of 88 patients demonstrated that prophylactic GJ decreased the incidence of late GOO and that a retrocolic GJ should be performed routinely for surgical palliation of unresectable periampullary carcinoma [6]. In yet another study of 65 patients who were found to be unresectable at exploration and underwent either a single bypass (HJ) or a double bypass (HJ and retrocolic GJ), prophylactic GJ decreased the incidence of GOO without increasing the rate of complications. There were no significant differences in the quality of life between the single- and double-bypass groups. The study recommended double bypass rather than a single bypass [7].

When comparing open GJ to laparoscopic GJ for the treatment of malignant GOO, several studies have favored laparoscopic GJ. A randomized controlled trial of 24 patients demonstrated a shorter time to oral intake and a lower rate of delayed gastric emptying with laparoscopic GJ [8]. In a retrospective review of 20 patients who underwent a palliative GJ for malignant GOO, no significant differences in surgical outcomes were noted between the open and laparoscopic GJ groups, although the study had a limited sample size [9]. When compared to surgical gastroenterostomy, endoscopic gastroenterostomy for malignant GOO has been associated with a decreased length of stay, shorter time to resume oral intake, and shorter time to resume chemotherapy, but increased reintervention rates [10]. In a study that compared surgical gastroenterostomy to endoscopic placement of enteral stents in 99 patients with malignant GOO and advanced pancreatic cancer, there were no significant differences in technical success, clinical success, adverse events, and survival between the two modalities, although enteral stenting resulted in an earlier return to oral intake and a shorter length of stay [11].

### 2.2. Interventional Radiology Palliation

**Figure 2:**
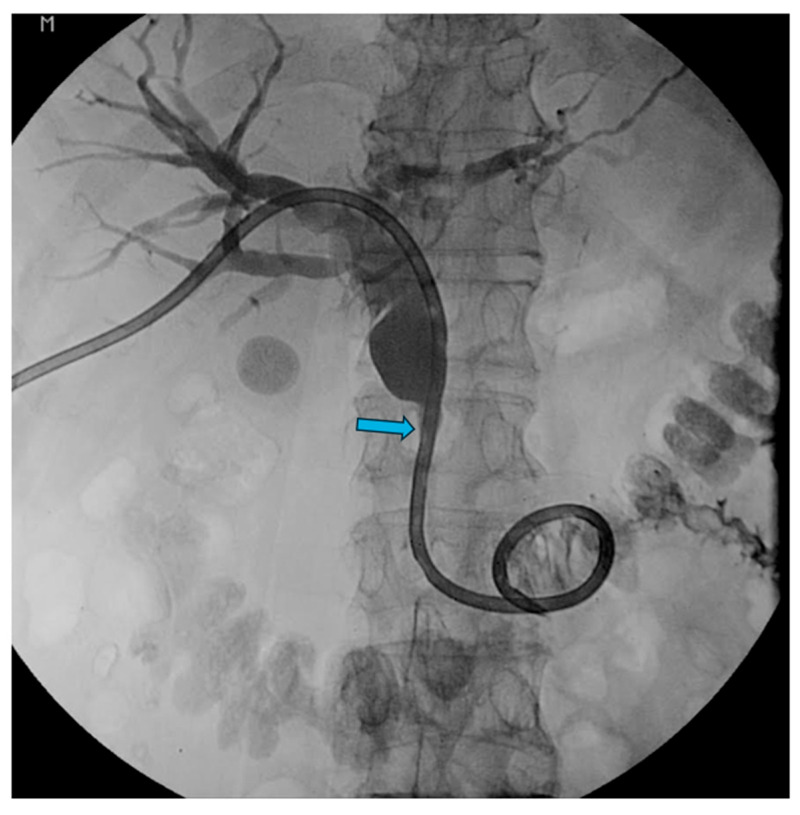
Fluoroscopy image reveals a percutaneous transhepatic biliary drain placed to bypass distal common bile duct obstruction due to unresectable pancreatic cancer (arrow).

**Figure 3:**
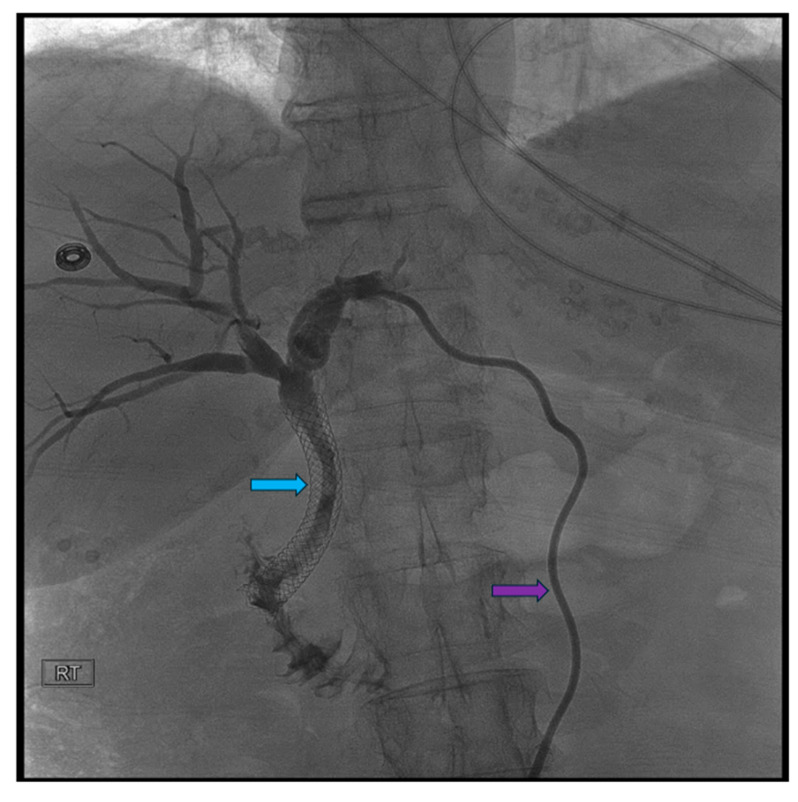
Fluoroscopy image reveals an uncovered self-expandable metal stent (blue arrow) inserted through a left-sided percutaneous transhepatic biliary drain tract (purple arrow). The percutaneous transhepatic biliary drain is typically capped and removed after confirming adequate biliary drainage through the self-expandable metal stent.

Non-surgical interventional therapies started with percutaneous transhepatic biliary drainage (PTBD), both as a palliative maneuver in unresectable patients with obstructive jaundice and to improve liver functions in patients whose surgery was delayed (Figure 2). PTBD, and insertion of plastic or self-expandable metal stents (SEMS) through the PTBD tract in some cases, was previously considered as the standard method for biliary drainage in patients with non-resectable malignant obstructive jaundice before being largely replaced by ERCP and biliary stent placement (Figure 3). PTBD remains a useful tool especially in patients with a large disease burden, those who are poor surgical candidates, or those who have had unsuccessful surgical or endoscopic drainage [12]. The technical success of PTBD has been reported to be close to 100%, whereas the clinical success rates may vary between 76.5% and 98% [13,14]. Approximately 20–25% of patients can develop complications, i.e., cholangitis, bleeding, catheter occlusion, or misplacement [13]. In a retrospective study of 16,822 patients who underwent PTBD for pancreaticobiliary malignancies, the 30-day mortality was 23.1%, especially in older men with increased comorbidities [15]. In a large retrospective study of 14,808 patients using the Surveillance, Epidemiology, and End Results–Medicare database comparing overall survival of patients with pancreatic cancer who underwent ERCP and those who underwent PTBD, ERCP was associated with a better mortality rate (adjusted hazard ratio [aHR] of 0.67; 95% confidence interval [CI] of 0.60–0.75) [16].

Percutaneous cholecystostomy (PC) has been shown to be an effective option for acute cholecystitis, especially in nonsurgical candidates [17,18]. PC has also been performed in patients who developed cholecystitis with malignant obstructive jaundice. In a case-control study of 206 patients with malignancies, PC was associated with a higher rate of acute cholecystitis resolution compared to antibiotics, with those with abdominal malignancies having an increased odds of resolution [19]. Additional studies are warranted to explore the role of PC in patients with unresectable pancreatic cancer.

### 2.3. Advanced Endoscopy Palliation

#### 2.3.1. Endoscopic Retrograde Cholangiopancreatography

An endoscopic approach has been the mainstay of palliation in patients with unresectable cancer since the 1990s. Endoscopic retrograde cholangiopancreatography (ERCP) and placement of plastic stents or self-expandable metal stents (SEMSs) has been used both preoperatively and palliatively. ERCP was initially introduced as a diagnostic modality in 1968 by Dr. William S. McCune, an obstetrician. It was not until 6 years later that an endoscopic sphincterotomy was performed, and it was 12 years before Soehendra et al. performed the first biliary stent placement in 1980, which opened the door to a less invasive treatment of malignant obstructive jaundice than palliative surgery or PTBD [20]. Since then, ERCP has become a diagnostic and therapeutic tool for a variety of hepatopancreaticobiliary pathologies.

While surgical and endoscopic biliary drainage have similar rates of technical success and efficacy, endoscopic biliary drainage is associated with fewer complications, improved quality of life, shorter hospital stay, and a lower cost. Endoscopic transpapillary stenting has become the preferred therapy of choice for patients with obstructive jaundice in the setting of unresectable pancreatic cancer, and high rates of improvement in jaundice and pruritus have been reported with transpapillary drainage.

Plastic stents were initially used for biliary drainage, although they tended to occlude several weeks or months after their placement. In a study of 49 patients with resectable or locally advanced pancreatic adenocarcinoma and biliary obstruction, approximately 27 out of 49 patients required repeat ERCP for stent exchange with a median of 82.5 days after original stent placement [21]. SEMSs were introduced in 1989 and have a larger diameter (10 mm) than plastic stents, which have a diameter of 7–10 Fr [21,22,23]. Multiple studies have compared plastic stents vs. SEMSs in patients with malignant biliary obstruction. A meta-analysis of 13 studies revealed lower rate of stent dysfunction (21.6% vs. 46.8%, *p* < 0.00001) and a lower rate of reintervention with SEMS (21.6% vs. 56.6%, *p* < 0.00001) compared to plastic stents, with no difference in complications [24]. Additionally, in the SEMS group, the mean survival rate was higher, the stent patency period was longer, and there was a lower cumulative cost per patient [24]. Although SEMSs, uncovered, partially covered, or completely covered, are initially more expensive than plastic stents, the total costs after 1 year are not significantly different between the two groups [25]. In a randomized controlled trial of 119 patients with pancreatic cancer on neoadjuvant therapy, covered and uncovered SEMSs were associated with similar rates of biliary drainage. Stent complications depended on stent type, stent length, and the presence of a gallbladder [26]. Plastic stents remain commonly used as they are relatively easy to place. Covered SEMSs have been associated with a higher rate of stent dysfunction from sludge, stent migration, and tumor overgrowth compared to uncovered or partially covered SEMSs, which have been associated with higher rates of tumor ingrowth.

The complication rate for ERCP in patients with malignant obstructive jaundice has been reported as 13% [27]. The most common adverse events of endoscopic transpapillary biliary drainage include pancreatitis, cholangitis, bleeding, perforation, cholecystitis, and liver abscess. The risk of bleeding has been demonstrated to be increased with sphincterotomy. In a retrospective study of 73 patients with pancreatic cancer, palliative biliary drainage did not have an effect on median progression-free survival and overall survival [28].

#### 2.3.2. Enteral Self-Expandable Metal Stents

**Scheme 1:**
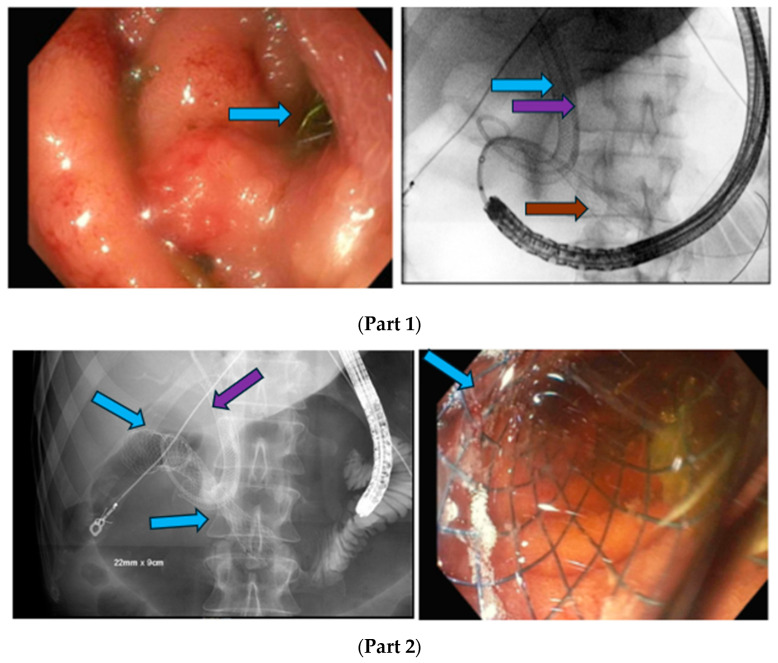
(**Part 1**). Endoscopic image reveals the biliary SEMS (**top left**, blue arrow) compressed by a malignant obstruction of the duodenum. Fluoroscopy image (**top right**) demonstrates a 7-French × 15 cm plastic stent (purple arrow) in a previously placed biliary SEMS (blue arrow). Note marked superior displacement of the biliary stents. The brown arrow demonstrates the placement of a duodenal SEMS in this patient with gastric outlet obstruction due to advanced pancreatic cancer. (**Part 2**). Fluoroscopy image (**left**) reveals a fully expanded, duodenal SEMS (blue arrows). The purple arrow demonstrates previously placed biliary stents. Endoscopic image (**right**) of a fully expanded, duodenal SEMS (blue arrow). Abbreviation: SEMS = self-expandable metal stent.

Additionally, endoscopic gastro-duodenal SEMSs have been utilized for GOO (Scheme 1 Part 1 and Part 2). In a prospective, multicenter study of 39 patients with malignant GOO, duodenal stenting had a technical success rate of 100% and clinical success rate of 92.3% [29]. In a study of 292 patients, among which 196 had pancreatic cancer and 96 patients had nonpancreatic cancer, who underwent stent placement for malignant GOO, the median survival post-stent placement was similar despite better overall survival in patients with nonpancreatic cancer [30]. Overall survival was reduced in patients with pancreatic cancer (13.7 vs. 17.1 months, *p* = 0.004) [28]. GOO was noted to be a marker for poor survival regardless of the type of malignancy. Factors which were associated with better post-stent survival in both groups included chemotherapy and the absence of distant metastasis. Stent dysfunction occurred in 7.7% of patients [31]. In a study comparing enteral stenting to surgical gastroenterostomy, there were higher rates of persistent nausea and vomiting and increased length of stay in the surgical GE group (*p* = 0.0102). In an RCT of 18 patients, endoscopic stenting was associated with a shorter median length of procedure, mean time for restoration of oral intake, and median hospital stay, although no statistically significant differences between the enteral stenting or gastrojejunostomy groups were noted with respect to morbidity, mortality, delayed gastric emptying, and clinical outcomes at the 3-month follow-up [31]. Complications occur in 2% to 12% of patients and include occlusion (most common) by tumor ingrowth or food bolus, migration, hemorrhage, perforation, and aspiration pneumonia.

#### 2.3.3. Endoscopic Ultrasound-Guided Therapies

Endoscopic ultrasound (EUS) was initially used to cytologically diagnose and stage pancreatic cancer. In a comparative study, EUS demonstrated a higher accuracy for staging of pancreatic malignancies compared to CT for T1, T2, and T3 tumors [32]. Several additional studies have revealed high levels of sensitivity, specificity, and accuracy of EUS compared to those of CT for pancreatic malignancy [33].

The role of EUS has recently evolved from a purely diagnostic technique to a complex, interventional modality. ERCP is often the first modality for biliary drainage in patients with obstructive jaundice. Biliary cannulation is dependent upon several factors including patient anatomy and the endoscopist’s expertise. In patients with pancreatic malignancy, biliary cannulation may be challenging due to the distortion of the ampulla, a malignant biliary stricture, which is difficult to traverse, or biliary or duodenal obstruction. Advanced cannulation techniques (i.e., needle knife pre-cut, double-guidewire, and pancreatic septotomy) may be required, but confer an increased risk of adverse events. EUS-guided biliary drainage (EUS-BD) has increasingly become a feasible and efficacious choice for obstructive jaundice. EUS-BD has also opened the door to interventions such as gallstone lithotripsy [34]. A meta-analysis of 6 randomized controlled trials of 577 patients comparing EUS-BD and ERCP-biliary drainage (ERCP-BD) demonstrated similar efficacy and safety. EUS-BD, however, was associated with a significantly lower risk of reintervention, post-procedure pancreatitis, tumor ingrowth/overgrowth, and reduced hospital stay [35].

#### 2.3.4. Lumen-Apposing Metal Stents

**Figure 4:**
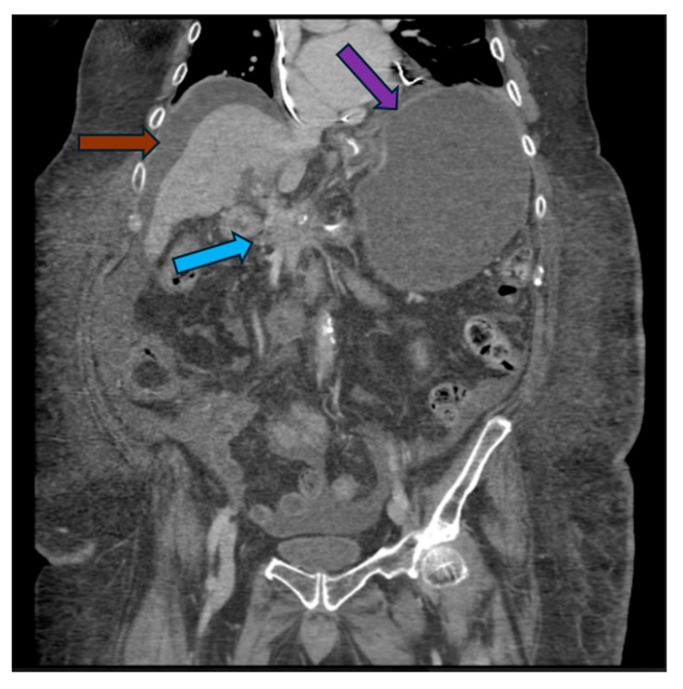
A coronal view of a computed tomography scan of the abdomen and pelvis showing an infiltrative pancreatic head mass (blue arrow) and a dilated, obstructed stomach (purple arrow). Ascitic fluid is demonstrated around the liver (brown arrow). Significant ascites usually precludes endoscopic ultrasound-guided gastroenterostomy.

EUS-guided placement of lumen-apposing metal stents (LAMSs) was first described in 2012 by Binmoeller and Shah who successfully performed a gastroenterostomy in a pig model [36]. Although LAMSs were initially developed for the use of peripancreatic fluid collections, they have been utilized in multiple gastroenterologic applications [37,38]. LAMS have allowed a myriad of palliative procedures, including cholecystoduodenal, choledochoduodenal, gastrohepatic, and gastroenteric anastomoses for cholecystitis, obstructive jaundice, and gastric outlet obstruction, respectively (Panels B–D, Figure 4) [39]. Electrocautery-enhanced delivery systems allow for direct access to the target lumen and have simplified the multi-step procedure of EUS-guided drainage. In a meta-analysis of 14 studies involving 620 patients who underwent EC-LAMS placement after a failed ERCP for malignant biliary obstruction (MDBO), the pooled rate of technical success was 96.7%, the pooled rate of clinical success was 91% and the rate of adverse events was noted to be 17.5% [40]. The overall re-intervention rate in the meta-analysis was 7.3% [40].

#### 2.3.5. Endoscopic Ultrasound-Guided Choledochoduodenostomy and Hepaticogastrostomy

EUS-guided choledochoduodenostomy (EUS-CDS) has also been demonstrated to be a promising technique for the management of MDBO. In a multicenter, randomized controlled trial of 144 patients with MDBO secondary to borderline resectable, locally advanced, or unresectable periampullary cancers, EUS-CDS was noted to be an efficient and safe alternative to ERCP with metal stent placement, although EUS-CDS was not superior with regard to stent function [41]. While it was traditionally performed if ERCP failed, EUS-CDS is now often performed as a first-line procedure for the drainage of MDBO [42].

**Scheme 2:**
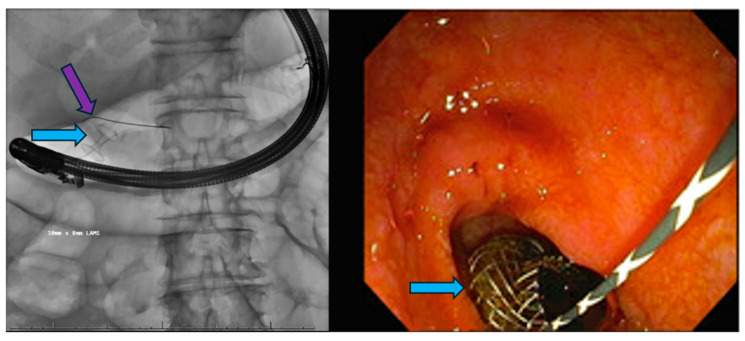
A fluoroscopic image (**left**) reveals an endoscopic ultrasound-guided choledochoduodenostomy using a 10 mm × 8 mm lumen-apposing metal stent (blue arrow) and a guidewire (purple arrow) in an air-filled, dilated common bile duct in a patient with a malignant distal biliary obstruction due to advanced pancreatic cancer. Endoscopic image (**right**) reveals the expanded, lumen-apposing metal stent with the guidewire in place (arrow).

**Scheme 3:**
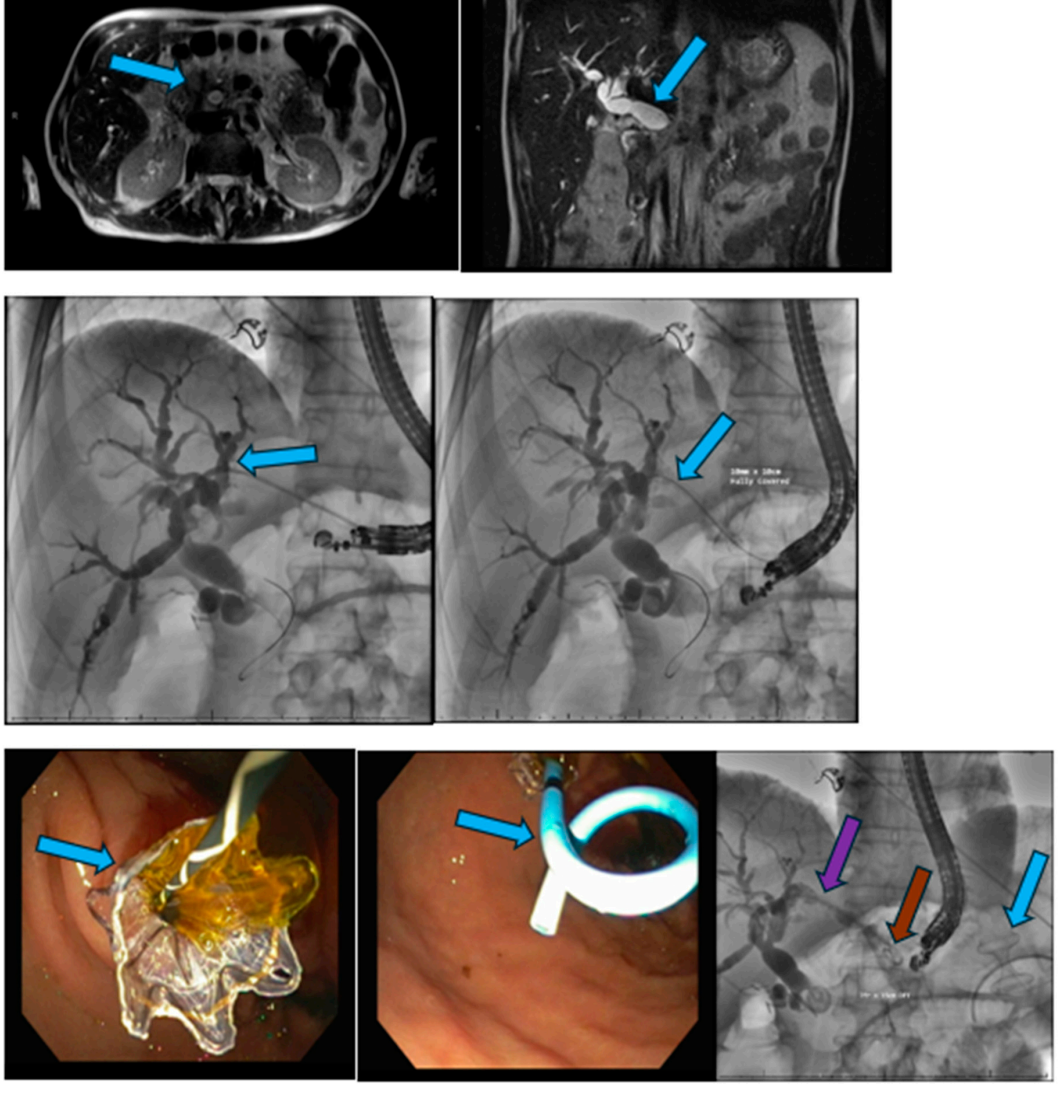
An axial view of a magnetic resonance cholangiopancreatography image (**top left** image) reveals a 2.1 cm pancreatic head mass (blue arrow) and marked intrahepatic and extrahepatic biliary dilation (**top right** image) with the common bile duct being approximately 1.98 cm in diameter (blue arrow). Fluoroscopy image (**middle left**) reveals puncture from the stomach to the left intrahepatic duct using a 19-gauge needle, a guidewire in place and an intraoperative cholangiogram with significant intrahepatic and extrahepatic biliary dilation. Fluoroscopy image (**middle right**, arrow) and endoscopy image (**bottom left**, arrow) reveals a 10 mm × 10 cm fully covered, self-expandable metal stent, which was inserted into the biliary tree to create an endoscopic ultrasound-guided hepaticogastrostomy. Subsequently, a 7-French × 15 mm double-pigtail stent was inserted in the self-expandable metal stent (**bottom middle** image, arrow) for biliary drainage. The bottom right image reveals the self-expandable biliary stent (purple arrow) and the double-pigtail stent (brown arrow). Additionally, a lumen-apposing metal stent was used to perform a gastroenterostomy due to gastric outlet obstruction (blue arrow). Image courtesy of Amar Vedamurthy, Division of Gastroenterology and Hepatology, Virginia Mason Franciscan Health, Seattle, WA, USA.

A network meta-analysis of 6 randomized controlled trials and 583 patients compared the effectiveness of EUS-CDS with LAMS [Scheme 2], EUS-CDS with SEMS, EUS-hepaticogastrostomy, [Scheme 3] ERCP, and PTBD performed upfront for the management of MDBO [43]. EUS-CDS with LAMS was associated with the highest rate of technical and clinical success and was noted to be significantly superior to ERCP as an upfront modality. Additionally, PTBD was associated with an increased risk of adverse events [43]. In a study of 14,808 patients with unresectable pancreatic cancer, patients who underwent ERCP had reduced mortality, shorter hospital stays, and lower hospital charges compared to patients who underwent PTBD. Both ERCP and PTBD were associated with improved survival of patients compared to those who did not undergo biliary intervention. PTBD involves an external drain which may impact patients’ quality of life [44]. EUS-guided gallbladder drainage compared to PTBD in patients who fail ERCP has been shown to be associated with decreased adverse events and to be more cost-effective due to requiring fewer reinterventions [45].

#### 2.3.6. Endoscopic Ultrasound-Guided Gastroenterostomy

**Scheme 4:**
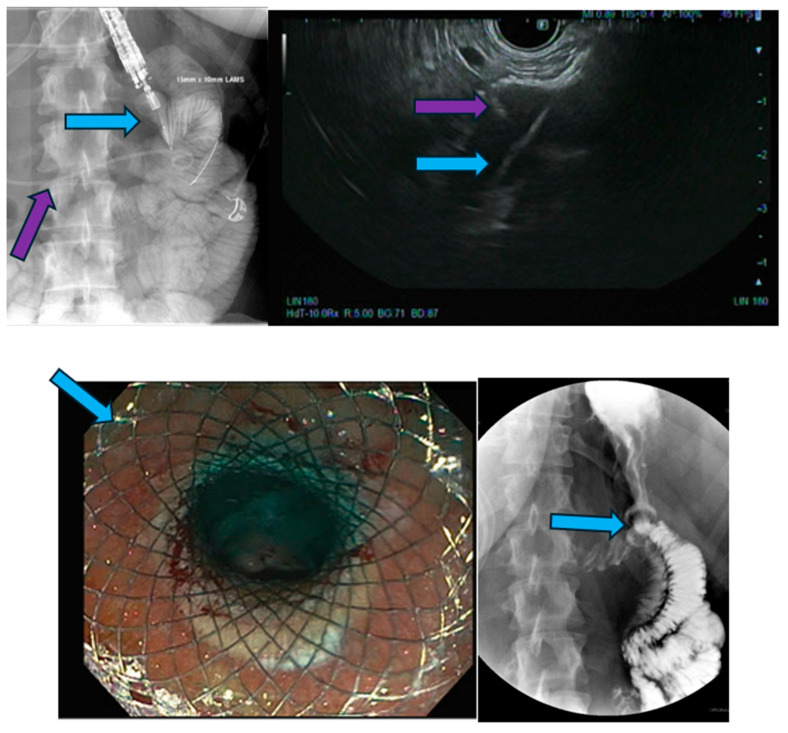
Fluoroscopy image [**upper left**] reveals a distal stent delivery of a 15 mm × 10 mm lumen-apposing metal stent (blue arrow) from the stomach to the small bowel. A nasobiliary drain (purple arrow) was used to instill contrast and methylene blue to localize and distend the small bowel. Endoscopic ultrasound image [**upper right**] reveals needle puncture (blue arrow) from the gastric wall to the small bowel. Note the delivery of the distal flange of the LAMS (purple arrow). Endoscopic image [**lower left**] of a fully deployed LAMS (blue arrow). Upper gastrointestinal series with oral contrast [**lower right**] demonstrates passage of oral contrast into the small bowel, which demonstrates stent patency (blue arrow).

While surgical gastroenterostomy and enteral stenting are the gold standard for the management of GOO, surgical GE is limited by its invasive nature and high morbidity rates, whereas enteral stenting is limited by stent patency duration and higher rates of re-intervention. EUS-GE was initially described in 2002 by Fritscher-Ravens in a porcine animal model [36,46,47]. EUS-GE was subsequently used in clinical practice following the development of LAMS. The pooled technical success rate and the pooled clinical success rate for EUS-GE have been reported to be 92% and 90%, respectively [48]. Several studies have demonstrated that while EUS-GE is comparable to SGJ, EUS-GE is associated with fewer associated adverse events, earlier resumption of diet, and a shorter hospital stay. EUS-GE has been noted to have a lower risk of obstruction and a decreased symptom recurrence compared to ES and in a recently published retrospective propensity score-matched study. EUS-GE has also been associated with fewer reinterventions, improved stent patency, and earlier oral intake compared to ES, although survival and patency of EUS-GJ and enteral stents were equivalent [49]. In a meta-analysis of 61 studies comparing enteral stenting to endoscopic or surgical GJ for malignant GOO, the clinical efficacy between the 3 groups was similar, although duodenal SEMSs were associated with a lower rate of procedure-related bleeding and a higher rate of reintervention [50]. The European Society of Gastrointestinal Endoscopy has recommended EUS-GE for malignant gastric obstruction as an alternative to enteral stenting or surgery in an expert setting [51] (Scheme 4).

#### 2.3.7. Endoscopic Ultrasound-Guided Gallbladder Drainage

Several studies have revealed that EUS-gallbladder drainage using LAMS is a safe and effective technique in patients with acute cholecystitis who are non-surgical candidates [52,53,54,55]. However, many studies were not restricted solely to patients with unresectable pancreatic cancer. 

In a retrospective study of EUS-gallbladder drainage using LAMS in patients with acute cholecystitis, a technical success rate of 94.8% and a clinical success rate of 100% were noted [52].

#### 2.3.8. EUS-Guided Celiac Plexus Neurolysis

**Figure 5:**
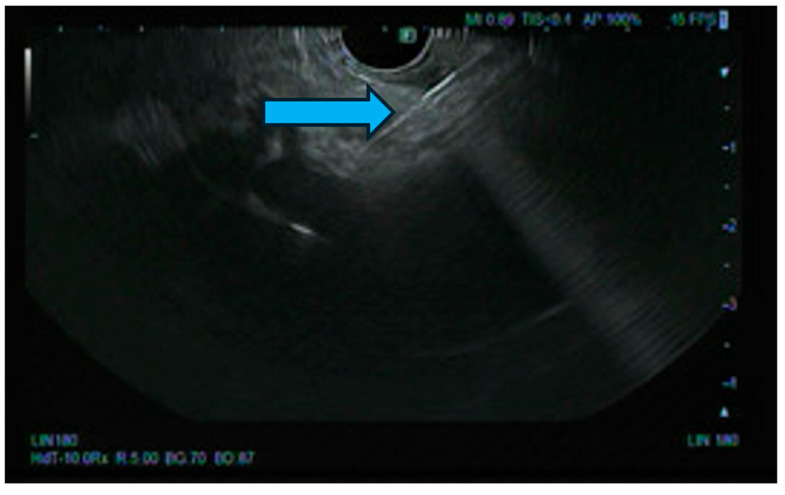
Endoscopic ultrasound image reveals needle puncture (arrow) from the gastric wall to the celiac plexus. Alcohol and local anesthesia were injected to provide neurolysis in a patient with advanced pancreatic cancer.

EUS has been utilized to perform a celiac block or ablation for palliation of unresectable pancreatic malignancy. EUS-guided celiac plexus neurolysis (EUS-CPN) involves the ablation of nerve tissue with the injection of alcohol and local anesthesia into the celiac plexus. The transgastric approach has been reported to be safer and more accessible compared to the percutaneous approach. EUS-CPN typically provides improvement in pain for 4–8 weeks. The efficacy of EUS-CPN varies from 50 to 94% in various studies [56] (Figure 5).

#### 2.3.9. Novel Endoscopic Ultrasound-Guided Therapies

EUS-guided RFA (EUS-RFA) is an emerging modality and has been reported to be a safe and efficacious technique in patients with advanced pancreatic cancer [57]. EUS-RFA may reduce tumor burden and improve the efficacy of chemotherapy [58,59].

EUS has also been employed in the placement of fiducials, which are radiographic markers used to define the borders of the pancreatic malignancy, and can improve the accuracy of target delineation in stereotactic body radiation therapy [60,61,62].

Finally, EUS-guided brachytherapy with radioactive iodine seeds has been reported to be efficacious in several studies [63]. In a study of 15 patients with unresectable pancreatic cancer, 30% of patients had a favorable response [64]. In a study of 8 patients with T4 pancreatic adenocarcinoma with a median follow-up period of 8.3 months, EUS brachytherapy was noted to be favorable, mostly due to decreased pain, in 4 out of 8 patients. No local complications were reported [65]. Treatment with iodine-125 seeds in patients with unresectable pancreatic cancer has also been demonstrated to prolong survival and improve biliary stent patency as well as improve patient quality of life by reduction in pain [66].

## 3. Conclusions

The palliation of unresectable pancreatic cancer has largely evolved from open or laparoscopic bypass of the bile duct or stomach in cases of obstructive jaundice or GOO, respectively, to minimally invasive therapies involving interventional radiology and advanced endoscopy. PTBD was initially utilized for patients with malignant obstructive jaundice and included the placement of plastic stents and, ultimately SEMSs, through PTBD tracts. Percutaneous cholecystostomy has also been used for the management of acute cholecystitis in the setting of malignant obstructive jaundice. Subsequently, ERCP with plastic stents or SEMSs, both preoperatively and palliatively, became the mainstay for obstructive jaundice, whereas endoscopic gastroduodenal SEMS were utilized for GOO. The adoption of EUS and LAMS allowed for a multitude of palliative procedures, including cholecystoduodenal, choledochoduodenal, gastrohepatic, and gastroenteric anastomoses for cholecystitis, obstructive jaundice, and gastric outlet obstruction, respectively. EUS-CPN has been demonstrated to be efficacious with regard to intractable pain in patients with unresectable pancreatic cancer. Additional EUS-guided therapies, including hepaticogastrostomy followed by SEMS placement for biliary decompression, radiofrequency ablation, fiducial placement, and radioiodine pellets, all serve as novel tools for palliation. Future innovations include artificial intelligence, which may assist with the early diagnosis of pancreatic cancer, risk stratification, personalized treatment plans, and predictions of overall survival [67,68]. Artificial intelligence can also prompt clinicians to engage in end-of-life planning and referral to palliative care in patients who are at a high risk for mortality. Robotic interventions for biliary and gastric outlet interventions have also been described for the palliation of unresectable pancreatic cancer [69]. Multidisciplinary collaboration, between oncologists, surgeons, interventional radiologists, advanced gastroenterologists, and primary care providers, is paramount to a successful outcome in the palliation of advanced pancreatic cancer.

## Data Availability

Not applicable.
